# Transformation of Selected *Fusarium* Toxins and Their Masked Forms during Malting of Various Cultivars of Wheat

**DOI:** 10.3390/toxins13120866

**Published:** 2021-12-04

**Authors:** Edyta Ksieniewicz-Woźniak, Marcin Bryła, Dorota Michałowska, Agnieszka Waśkiewicz, Tomoya Yoshinari

**Affiliations:** 1 Department of Food Safety and Chemical Analysis, Prof. Waclaw Dabrowski Institute of Agricultural and Food Biotechnology—State Research Institute, 02-532 Warsaw, Poland; marcin.bryla@ibprs.pl; 2 Beer and Malt Laboratory, Prof. Waclaw Dabrowski Institute of Agricultural and Food Biotechnology—State Research Institute, 02-532 Warsaw, Poland; dorota.michalowska@ibprs.pl; 3 Department of Chemistry, Faculty of Forestry and Wood Technology, Poznan University of Life Sciences, 60-625 Poznan, Poland; agnieszka.waskiewicz@up.poznan.pl; 4 Division of Microbiology, National Institute of Health Sciences, Kawasaki-ku, Kawasaki-shi, Kawasaki 210-9501, Kanagawa, Japan; t-yoshinari@nihs.go.jp

**Keywords:** malting, wheat, modified mycotoxins, *Fusarium* toxins, biotransformation

## Abstract

This study investigated the impact of malting of six wheat cultivars inoculated with *Fusarium culmorum* on the dynamics of content changes of selected *Fusarium* toxins. The grains of all the tested cultivars showed a high content of deoxynivalenol (DON), zearalenone (ZEN), and their derivatives, whereas nivalenol (NIV) and its glucoside were found only in the Legenda cultivar. Our experiments confirmed that the malting process of wheat grain enables the secondary growth of *Fusarium*, and mycotoxin biosynthesis. The levels of toxins in malt were few-fold higher than those in grain; an especially high increase was noted in the case of ZEN and its sulfate as the optimal temperature and pH conditions for the biosynthesis of these toxins by the pathogen are similar to those used in the grain malting process. This is the first paper reporting that during the malting process, biosynthesis of ZEN sulfate occurs, instead of glycosylation, which is a typical modification of mycotoxins by plant detoxication enzymes.

## 1. Introduction

Wheat has been one of the most widely grown crops in the world since the dawn of humankind. In addition to the basic food products made from wheat (such as bread, pasta, and confectionery products), wheat grain is also a raw material used to produce malt, and consequently beer. According to the latest version of the German Beer Purity Law and the World Beer Cup Style Guidelines, wheat beer must be top-fermented and made with at least 50% malted wheat [1]. In recent years, the growing demand for craft beers has resulted in an increased demand for wheat malts, which has led to the search for appropriate materials for their production [2]. Although wheat is the world’s second-largest crop after maize, there is still a shortage of suitable varieties for the generation of malt that could be used later in beer production. The high protein content, which is desired by farmers and bakers, is a negative factor for brewers as it can lead to many problems in the production process (e.g., extended rinsing time, filtration difficulties, reduced flavor stability of the product) [3].

Malting is associated with a rapid increase in the water content of the grains during the washing and steeping stages. Such conditions favor the growth of microorganisms that are naturally present on the grain, which may have an adverse effect on the physicochemical properties of the malt, in addition to posing a risk to the safety of the final product [4]. The most important fungal pathogens of cereals are *Fusarium* spp., which contribute to huge losses in cereal production, and their secondary metabolites commonly known as mycotoxins, which pose a threat to human and livestock health [5,6]. The most important *Fusarium* toxins include trichothecenes (deoxynivalenol (DON), nivalenol (NIV), HT-2 toxin, T-2 toxin], zearalenone (ZEN), and fumonisins (FBs)). The harmful health effects of these toxins include neurotoxicity, nephrotoxicity, hepatotoxicity, genotoxicity, immunotoxicity, potential carcinogenicity, and estrogenic or teratogenic effects [7].

The presence of mycotoxins activates plant metabolic detoxification pathways leading to the formation first- and second-phase metabolites, which accumulate in vacuoles or are bound to cell walls [8]. Changes in the structure of mycotoxins can also be caused by the activity of some microorganisms, such as bacteria or fungi. The modified forms of mycotoxins are often referred to as “masked” forms, due to the impossibility of quantifying them with methods used for the analysis of their primary forms [9,10]. The direction and rate of mycotoxin transformations may therefore depend on the initial toxin content, the presence of pathogenic strains capable of their biosynthesis, and the enzymatic activity of the malt [11].

The aim of this study was to determine the variability in the biosynthesis of selected *Fusarium* mycotoxins during the malting process of different cultivars of wheat grain. In addition, the fungi growth during the entire process was investigated by ergosterol analysis. To date, there has been limited knowledge regarding the transformation of mycotoxins during the malting process. Our preliminary work highlighted the possible hazard of secondary fungi growth during beer malting [12], while this work investigated these processes in detail. To the best of our knowledge, this study is one of the first to describe the transformation processes of mycotoxins, including their metabolites, during the malting of wheat grain.

## 2. Results and Discussion

### 2.1. “In Vitro” Evaluation of the Toxicity of Fusarium culmorum KF-846 Strain

*F. culmorum*, which was used in the experiments, was found to be able to biosynthesize mycotoxins. The mycotoxin profile of this strain was as follows: ZEN 290 ± 11 μg/g, ZEN-14S (zearalenone-14-sulfate) 2600 ± 174 μg/g, α-ZEL (α-zearalenol) 3.1 ± 0.2 μg/g, β-ZEL (β-zearalenol) 6.5 ± 1.1 μg/g, and DON 30.2 ± 2.6 μg/g, while NIV was not detected. ZEN-14G (zearalenone-14-glucoside), DON-3G (deoxynivalenol-3-glucoside), and NIV-3G (nivalenol-3-glucoside), being plant metabolites of the mycotoxins, were also absent in the sample.

As it is known from the literature, the intraspecific variability of *F. culmorum* isolates is exceptionally large and depends on many factors [13,14]. It is also worth mentioning that in vitro tests may not be suitable for predicting the efficiency of mycotoxin biosynthesis under field conditions because the course of infection depends on the genotype of the plant as well as on climatic conditions, which cannot be controlled.

As demonstrated in our research, the applied strain was characterized by a high biosynthetic capacity for ZEN-14S (higher than that of ZEN). There is still a lack of data on ZEN-14S biosynthesis, and the scope of the above-mentioned research did not address these aspects. In the available literature, there are only fragmentary data on ZEN-14S biosynthesis, for example, Plasencia and Mirocha showed that the ability of *Fusarium* to biosynthesize ZEN-14S depends on the species and strain of *Fusarium*, but its content was lower than that of ZEN (in a ratio of 12:1 to 2:1) [15].

### 2.2. Changes in the Ergosterol Content during the Malting of Wheat Grain

The presence of fungal spores in wheat grain, in addition to favorable conditions, enabled the secondary growth of the pathogen, which was observed by means of a macroscopic evaluation. The development of the pathogen during malting was also confirmed by conducting ergosterol (ERG) analysis in the samples. ERG is the main sterol used by fungal cells to control the fluidity of their cell membranes. It does not occur naturally in plant and animal cells, so, according to some authors, its presence in grain samples can be quantitatively associated with a fungal contamination and mycotoxin levels [16,17,18]. The determined ERG content in the starting material (wheat grain) ranged from 14.6 ± 0.5 mg/kg (Pokusa cultivar) to 44.8 ± 1.3 mg/kg (Muszelka cultivar). After steeping the grain, the ERG content ranged from 8.2 ± 0.4 mg/kg (Pokusa cultivar) to 35.5 ± 1.0 mg/kg (Muszelka cultivar), and its content in grain samples significantly decreased, ranging from 21% (Muszelka cultivar) to 56% (Legenda cultivar). During wheat grain malting, a significant increase was observed in the ERG content in the tested samples. At the end of the malting stage, the ERG content was significantly higher than in the initial stage and ranged from 20.9 ± 1.4 mg/kg (Sailor cultivar) to 69.9 ± 3.7 mg/kg (Muszelka cultivar). In most of the analyzed cases, the ERG contents in the grains derived from the inoculated wheat were similar to those described in the literature. In the study of Lamper et al., the observed ERG content ranged from 1.20–42.14 mg/kg with a relatively high DON content (0.08–33.71 mg/kg) [16], while Miller, Young, and Sampson found the ERG content in wheat to be at the level of 5.16–18.2 mg/kg, with the DON content in the grain at the level of 230–9540 µg/kg [19]. Dohnal et al. reported that the ERG content in barley grain rose from 0.88–15.87 mg/kg to 2.63–34.96 mg/kg after malting, but the DON content in the malt was relatively low at a maximum level of 641 µg/kg [20]. Schwarz, Casper, and Beattie identified a significant increase in the ERG content during the malting of barley grains naturally infected with the pathogen *Fusarium* [21].

### 2.3. Changes in the Trichothecenes Content during the Malting of Wheat Grains

Prior to the malting process, six wheat grain samples of different cultivars obtained from spikes inoculated with *F. culmorum* were analyzed for their mycotoxin content. Inoculation of the wheat plants with the spores of the pathogen resulted in the appearance of symptoms of an infection, such as a characteristic whitening of the ear. The mycotoxin levels in the tested samples were significant, but it should be noted that the potential for mycotoxin biosynthesis may be limited due to unfavorable weather conditions for the development of the pathogen infection in plants during heading and flowering. All results of the mycotoxin quantification are presented in Table 1. The DON content ranged from 2.49 ± 0.23 mg/kg for the Pokusa cultivar to 9.59 ± 0.50 mg/kg for the Muszelka cultivar. The presence of NIV (>LOQ) was found only in the Legenda cultivar (0.90 ± 0.03 mg/kg). ZEN ranged from 0.13 ± 0.02 mg/kg (Legenda) to 0.79 ± 0.01 μg/kg (Muszelka). There was no presence of α-ZEL in any of the six wheat samples, while β-ZEL was present at a relatively low level from 10.3 ± 2.1 μg/kg (Legenda) to 62.2 ± 7.1 μg/kg (Muszelka). The content of ZEN-14S was estimated to be in the range of 0.60 ± 0.05 mg/kg (Sailor) to 3.64 ± 0.17 mg/kg (Muszelka). NIV-3G, DON-3G, and ZEN-14G were also analyzed in the samples. ZEN-14G was not found in any of them, while the content of DON-3G ranged from 0.43 ± 0.06 mg/kg (Legenda) to 1.24 ± 0.13 mg/kg (Muszelka). NIV-3G coexisted with NIV, and its content was 0.31 ± 0.01 mg/kg.

In the case of the elevated level of ZEN-14S, the lack of literature data in this field makes it impossible to have a detailed discussion; nevertheless, it should be emphasized that the strain used has a high potential for the biosynthesis of ZEN-14S, in the natural environment as well, which can be particularly important in the context of food safety.

ANOVA showed that the malting process had a significant impact on the mycotoxin content. The direction and speed of mycotoxin production and transformation differed depending on the cultivar, but the general tendency was similar: steeping decreased the content of mycotoxins, while malting caused an increase in the same. Table 2 shows the relative changes in the mycotoxin content during the malting process.

Steeping decreased the DON content to levels in the range of 1.29 ± 0.18 mg/kg (Tonacja) to 4.11 ± 0.38 mg/kg (KWS Ozon), which was a decrease in the range of 27% (Pokusa) to 72% (Tonacja) (Table 2). From the first day of malting, a gradual increase was observed in the mycotoxin content. In the case of DON, its content increased during the seven-day malting compared to the grain after steeping at the range from 188% (KWS Ozon) to 649% (Legenda). Thus, after the malting process, the DON content in the malt increased and it was at the range from 6.53 ± 0.92 mg/kg (Pokusa) to 11.84 ± 1.33 mg/kg (KWS Ozon). In all the test samples of the finished malts, the DON content exceeded the initial level. Compared to the grains, the DON content in the malt increased at the range from 23% (Muszelka) to 163% (Pokusa).

Several studies have reported an increase in the mycotoxin content during malting of wheat grain. This increase has been found to be highly dependent on grain characteristics and malting technology. Jin et al. malted 15 samples of a red spring wheat with initial DON levels of 200–10,130 μg/kg. They observed an average increase in the DON content during malting, which was 460% of the DON content in the grain [22]. In another experiment, the same authors reported 13 samples of winter wheat malt with a DON content of 107–995 µg/kg and observed an increase in the level of DON at the range from 18% to 568%, depending on the initial content of this compound in the wheat grain [23]. In a study by Dohnal et al., 20 samples of naturally contaminated barley were malted, and it was found that in malt, there was an average 32% increase in the DON content compared to the grain. It should be noted that, in this study, the level of contamination of DON barley grain was relatively low and met the requirements of the maximum permissible content in the European Union [20]. Lancova et al. conducted malting of a naturally (12 µg/kg) and artificially (238 µg/kg) DON-contaminated barley grain, derived from plants inoculated with a mixture of *F. culmorum* and *F. graminearum* spores (at a ratio of 1:1). The authors observed increases in the DON content by 275% and 114%, respectively, relative to the grain used in the malting process [24]. Zachariasova et al. observed an increase in the DON content during malting of barley grain (from 2467 µg/kg in barley grain to 11,638 µg/kg in finished malt). Schwarz et al. found that steeping of barley grain samples (with DON contents between 4.8 and 22.5 mg/kg) reduced the DON content. However, during the entire malting period, mycelial growth and de novo DON biosynthesis were observed [25].

Despite the inoculation of all wheat cultivars with the same pathogen, NIV was identified only in the Legenda cultivar. Steeping the grains resulted in a decrease in the NIV content from 0.90 ± 0.03 to 0.52 ± 0.07 mg/kg (42%), with subsequent malting days resulting in a gradual increase in the NIV content to 1.20 ± 0.10 mg/kg on the 7th day of malting, which is a 130% increase compared to the grain after steeping. The increase in the NIV content during the malting process, in contrast to DON, was not very fast. In the literature, there are scarce data regarding the changes in the NIV content during the malting process. An earlier study by Bryła et al. reported comparable results. Upon steeping of wheat grain containing 702 µg/kg of NIV, its content decreased by 26%, while during the malting process, its content increased, reaching a level comparable to the initial content [12]. Malachova et al. analyzed changes in the contents of selected toxins (including NIV) during barley grain malting. The authors showed that during the malting process, the content of the analyzed mycotoxins increased after the process in 70% of the samples, while it should be noted that each mycotoxin was present in the barley grain at a level below 50 µg/kg, indicating a low level of grain infection with pathogen spores or a low toxicogenic potential contributing to de novo biosynthesis of the mycotoxins [26]. Based on the results presented in this paper and previous research [12], it can be concluded that there is a slower biosynthesis trend of NIV during the malting process of wheat grain as compared to that of DON, and its content in malt may slightly exceed its content in the wheat grain. However, it should be mentioned that apart from the conditions of the malting process of cereal grains, the de novo biosynthesis ability of NIV may depend on a chemotype of a pathogen strain, which may determine the biosynthesis intensity.

**Figure 1:**
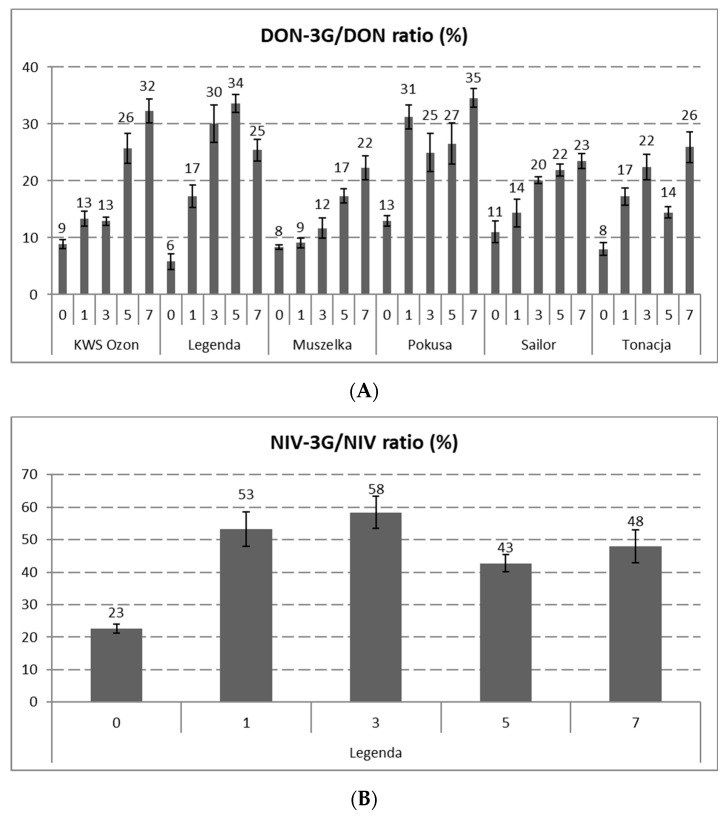
DON3G/DON (**A**) and NIV3G/NIV (**B**) molar ratios (%) at distinct stages of the malting process of grain of six wheat cultivars. (The days of malting: “0”—raw grain, “1”—grain after steeping, “3”—grain after 48 h of germination, “5”—grain after 96 h of germination, “7”—grain after 144 h of germination (finished malt). Abbr.: DON—deoxynivalenol, DON-3G—deoxynivalenol-3-glucoside, NIV—nivalenol, NIV-3G—nivalenol-3-glucoside. Error bars represent standard deviations of the values.

In case of DON-3G, there were no significant changes in its content after the process of steeping in some cultivars (KWS Ozon, Legenda, Sailor). In the Muszelka and Tonacja varieties, a significant decrease was observed in the DON-3G content, reaching the levels of 0.48 ± 0.01 and 0.35 ± 0.06 mg/kg, respectively (which represents a decrease of 61% and 40%), while in the Pokusa cultivar, a significant increase to a level of 0.88 ± 0.10 μg/kg (an increase of 77%) was observed. The possible explanation is a difference in the ratio of DON synthesis and its subsequent glycosylation on the one hand and the transfer of DON-3G to water on the other. As in the case of DON, a relatively high increase in the DON-3G content was observed during malting (especially in the final stage of malting), and the dynamics of the metabolite biosynthesis rate were higher than those of DON. The content of DON-3G after seven days of malting reached levels at the range from 3.04 ± 0.31 (Sailor) to 5.92 ± 0.12 mg/kg (KWS Ozon), which constituted an increase at the range from 296% (Pokusa) to 999% (Legenda) in relation to the grain after steeping. In turn, the increase in the DON-3G content in relation to unprocessed grain ranged from 229% (Muszelka) to 912% (Legenda). Moreover, it should be noted that an increasing share of the DON-3G content was observed during the malting process. At the beginning of malting, in unprocessed wheat grain, the relative content of DON-3G (in relation to DON) was in the range of 6% to 11% (depending on the grain cultivar). In the case of grain, after the malting process, this content ranged from 25% to 35% (Figure 1).

In the case of NIV-3G (found only in the Legenda cultivar), the process of steeping the grain did not significantly reduce its content. Nevertheless, there was an increase in the content of this substance on days following malting of the wheat grains, from 0.31 ± 0.01 mg/kg in the grain after steeping to 0.87 ± 0.07 mg/kg on the 7th day of malting (an increase of 183%). Similar to DON-3G, in the case of NIV-3G, there was an increase in the relative content of NIV-3G/NIV from 23% in wheat grain to 43–58%, depending on the malting day (Figure 1).

There is limited literature on the changes in the content of DON and DON-3G in malted wheat grains. In the case of NIV and NIV-3G, it is practically absent. It should be noted that the grain malting process caused a greater increase in the concentration of DON-3G from the grain steeping stage, as compared to DON. This observation is probably the result of the glucosyltransferase activity during grain malting, when second-phase metabolites of mycotoxins may be formed. The upward trend in the content of DON and NIV metabolites indicates that the life processes of the grain (macroscopic germination) were visible past the first day after grain steeping. To date, little is known about the dynamics of the biochemical reactions involving mycotoxins. Partially convergent results have been reported by Habler et al., who found that the increase in the content of DON-3G after steeping the grain was about 2–2.5 times higher than the content in the grain before steeping, and the malt obtained in this process contained more DON-3G than DON [27]. In our previous studies, significant increases in the contents of DON and NIV metabolites (compared to the initial stage) were found from the third, fourth, or fifth day of malting (depending on the wheat cultivar and type of analyte). On the last day of malting, the content of DON-3G increased by over 500% (Legenda) and 300% (Pokusa) compared to the grain after steeping, whereas in the case of NIV-3G, this increase exceeded 200% [12]. As in the previous studies, it was found in the experiment described here that significant increases in the content or an upward trend in the content of DON and NIV metabolites in malted wheat grain started earlier in the process than in the case of parent toxins. In our opinion, the ability to biosynthesize DON and NIV metabolites by the germinated wheat grain is mainly dependent on the quality of the grains used in the malting process. Grains having higher germination energy and containing a low proportion of damaged kernels (reduced degree of grain infection by *Fusarium*) may be characterized during malting by high activity of glucosyltransferases responsible for de novo DON-3G and NIV-3G biosynthesis. Therefore, different initial contents of mycotoxins in cereal grains (which may indicate the degree of grain infection) may determine the different course of the dynamics of the mycotoxin metabolite biosynthesis. In the experiment, the percentage content of DON-3G and NIV-3G was assessed, both in the starting material (wheat) and in the malted grain samples from a particular malting period, in relation to the basic analogues of these mycotoxins. The increase in the relative content of DON-3G and NIV-3G in the samples of malted wheat grain compared to the content of these substances in unprocessed grains demonstrates the importance of the malting process in the context of the presence of these metabolites in malt. Similar observations have been reported in previous studies [12]. The content of DON-3G in wheat grain was found to be in the range of 10–17%, while in malted wheat grain, this content was 19–55% of the DON content (depending on the wheat genotype and malting time) and stabilized between 4 and 7 days of malting. However, with respect to NIV-3G, the content in wheat was 22% of the NIV content and increased during malting, reaching the value of 27–99% of the NIV content [12]. Other studies indirectly indicated a relatively high content of DON and NIV metabolites. Ksieniewicz-Woźniak et al. found the DON-3G content in samples of the finished barley malt to be in the range of 22–186% of the DON content. Analogically, NIV-3G was in the range of 32–126% of NIV [28]. Similarly high DON-3G/DON values were described by Habschied et al., in barley malt, and depending on the method of malting and the level of *F. graminearum* infection, they obtained a relative content of DON-3G in the range of 45–82% [29]. Increased relative levels of DON-3G and NIV-3G metabolites have also been observed in many beer samples, which may confirm the high activity of glucosyltransferases during the malting process of grain, thus supporting the fact that there is secondary biosynthesis of DON-3G and NIV-3G [24,28,30,31].

### 2.4. Changes in the Content of ZEN and Its Modified Forms during Malting of Wheat Grain

For all wheat genotypes, the malting of wheat grains had a significant effect on the content of ZEN and its metabolites. Steeping the grains decreased the ZEN content in all test cases to levels at the range from 24 ± 3 (Legenda) to 208 ± 8 µg/kg (KWS Ozon), which constituted changes at the range from 51% (Pokusa cultivar) to 84% (Tonacja cultivar). However, the malting process resulted in a gradual significant increase in the ZEN content as compared to the content in the grain after steeping. The ZEN content in the malt on the last day ranged from 0.83 ± 0.03 (Legenda cultivar) to 9.11 ± 1.09 mg/kg (Muszelka cultivar); thus, its content increased (compared to grain after steeping) depending on the cultivar, from over 1600% for the Pokusa cultivar to almost 6000% for the Tonacja cultivar. Depending on the cultivar, the ZEN content in the finished malt was higher by 538–1059%, as compared to the raw grain.

**Figure 2:**
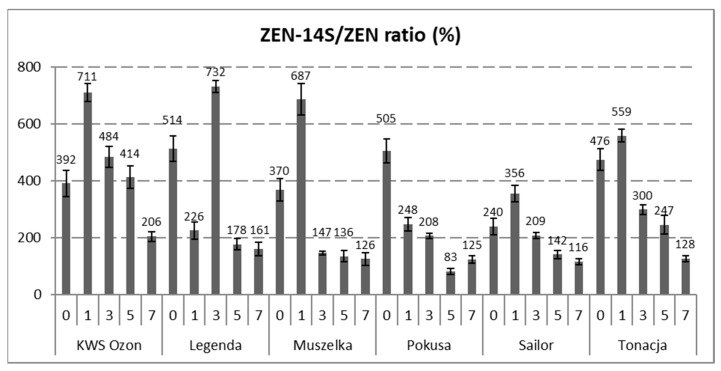
ZEN-14S/ZEN molar ratio (%) at various stages of the wheat grain malting process. The days of malting: “0”—raw grain, “1”—grain after steeping, “3”—grain after 48 h of germination, “5”—grain after 96 h of germination, “7”—grain after 144 h of germination (finished malt). Abbr.: ZEN—zearalenone, ZEN-14S—zearalenone-14-sulfite. Error bars represent standard deviations of the values.

The content of ZEN-14S in the wheat and malted grain samples was higher than that of ZEN. Similarly to ZEN, the content of ZEN-14S decreased during steeping grain at the range from 0.07 ± 0.01 (Legenda) to 1.85 ± 0.05 mg/kg (KWS Ozon). Thus, depending on the grain cultivar, the content of ZEN-14S decreased at the range from 48% (Sailor) to 92% (Legenda). As in the case of ZEN, the malting process was a time of an intensive increase in the content of ZEN-14S, with the growth rate dynamics being lower than in the case of ZEN. After the malting process, the content of ZEN-14S ranged from 1.66 ± 0.12 (Legenda) to 14.35 ± 0.88 mg/kg (Muszelka). The dynamics of the increase in the toxin content (percentage) compared to the grain before the steeping process ranged from 100 (Legenda) to 339% (Sailor). The differences in the dynamics of the rate of the ZEN and ZEN-14S biosynthesis were the reason for the change in the relative content of ZEN-14S (in relation to ZEN) on individual days of the malting process. A decreasing trend in the relative content of ZEN-14S was observed for all varieties of malted wheat grains. The estimated relative content of ZEN-14S in wheat grain ranged from 240 to 514%, while after the malting process, this content ranged from 116 (Tonacja cultivar) to 206% (KWS Ozon cultivar) (Figure 2).

β-ZEL was also detected in the samples, but its content was much lower than those of ZEN and ZEN-14S. As in the case of other derivatives, a decrease in the content of β-ZEL was observed after the steeping process, and in the case of three wheat cultivars, the content of this substance decreased to a value below the limit of quantification. However, during grain malting, an increase in the β-ZEL content was noted. Compared to the grain before the steeping process, the content of this substance increased to levels at the range from 19.9 ± 2.1 (Legenda) to 181.9 ± 28.6 µg/kg (Muszelka) (the range from 62% for the Tonacja cultivar to 192% for the Muszelka cultivar).

There is scarce literature on ZEN biosynthesis by pathogens during the malting process of cereal grains. During this process, a high increase in the ZEN content was observed after the grain steeping process, much higher than those observed in the case of trichothecenes. In our opinion, this is due to the fact that there is a difference in the optimal conditions for the biosynthesis of ZEN and trichothecenes (including DON). It is assumed that the optimal substrate pH and incubation temperature for DON biosynthesis are approximately 5 and 20–28 °C, whereas in the case of ZEN, approximately 9 and 15 °C, respectively [32,33,34]. In our study, the malting process was conducted at a temperature close to that optimal for ZEN biosynthesis by the pathogen. However, it should be emphasized that particular species and even strains of *Fusarium* genus may differ in the biosynthesis of this mycotoxin. In fact, studies on the effect of the malting process of cereal grains on the ZEN content differ in terms of the obtained results. According to Kocić-Tanackov et al., steeping of two samples of barley grain (naturally contaminated with ZEN at levels of 9.2 and 9.7 µg/kg) increased the ZEN content to 86.5 and 37.4 µg/kg, respectively (a different trend as compared to our research). During the complete process, the content of ZEN in malted grain was variable (it decreased during germination to the levels of 12.5 and 26.8 µg/kg) and increased after drying to the levels of 62.9 and 71.2 µg/kg). In the finished malt (after germination), the ZEN content was 35.7 and 17.8 µg/kg [35]. On the other hand, different results were obtained in the studies by Pascari et al., who upon malting two naturally contaminated barley grains (with initial ZEN content of 38 and 55 µg/kg) and three samples of barley grains infected in the laboratory with the pathogen (ZEN content in the range of 34 to 194 µg/kg) did not achieve significant changes in the content in each of the malting stages (although after the steeping process, a downward trend in the ZEN content was observed in barley samples naturally contaminated with ZEN, i.e., −48% and −72%). It should be mentioned that the malting process itself was conducted in a much shorter time (96 h), which may also have an impact on the obtained results [36]. The variability in the obtained findings may be an effect of the different conditions of the malting process, type of grain used, the concentration of fungal spores, and their strains, which may show different abilities to biosynthesize ZEN in different conditions. All these factors simultaneously affect the technological process and may contribute to the discrepancies in the obtained results. The above-mentioned factors and limited literature in this topic make the discussion of the obtained results difficult. The lack of literature data on the possibility of biosynthesis of modified ZEN derivatives during the malting process of cereal grain makes it troublesome to discuss the results. As time passed during the malting of the wheat grain, the rate of ZEN biosynthesis exceeded that of ZEN-14S (although the content of ZEN was lower than that of ZEN-14S). Despite this, significant increases in the ZEN-14S content were observed during this process. Discrepancies in the biosynthesis of these toxins by the pathogen may be due to the specific properties of the *Fusarium* strain used in inoculation (as also confirmed by the in vitro studies on rice) and several other factors. The complexity of this problem allows us to hypothesize that the pathogen during its growth in the technological malting process of grain can biosynthesize ZEN. However, due to its accumulation, *Fusarium* converts ZEN into a phase II metabolite through sulfation reactions. Unfortunately, there is still much to be discovered in this respect, especially the characterization of factors and their differences in the biosynthesis of ZEN and ZEN-14S by *Fusarium*. Although the grains used in this study came from wheat plants inoculated with the pathogen, the content of ZEN-14S in the wheat grain (higher than ZEN) as well as the rapid increase in the content of this substance during the malting of wheat grain is worrisome from the point of view of food and feed safety. Therefore, there is a need for further studies with a wider scope.

## 3. Conclusions

The present research confirmed that the malting process of wheat grains enables the secondary development of fungi, and the increases of toxin concentrations during the process are probably due to biosynthesis by *Fusarium* species present in grain. Greater increases in the content of DON-3G and NIV-3G in the initial malting time of the wheat grains (as compared to DON and NIV) may indicate secondary biotransformation processes of DON and NIV to their glycosidic forms with the participation of endogenous plant enzymes responsible for detoxification processes. In contrast, the highest increases in DON and NIV contents were observed in the final period of grain malting. The toxicogenic characteristics of the pathogenic strain of *F. culmorum* used for inoculation of the wheat plants showed the ability of this strain to biosynthesize ZEN-14S with surprisingly higher efficiency as compared to ZEN. This trend was also observed in the analyzed samples of the wheat grain obtained from plants inoculated with this strain. Malting of wheat grain resulted in a much higher percentage increase in the contents of ZEN and ZEN-14S than in those of trichothecenes. In our opinion, this may be due to the fact that the malting process was conducted in conditions close to optimal for the biosynthesis of ZEN by the fungus. Differences in the rate of biosynthesis were also observed between ZEN and ZEN-14S, which resulted in a decreasing trend of the relative ZEN-14S/ZEN content during the process. However, despite the higher efficiency of ZEN biosynthesis than ZEN-14S during grain malting, the ZEN content in samples after malting was lower than in the case of ZEN-14S. It must be noted that the data obtained using highly contaminated grain cannot be simply extrapolated to lower levels of contamination, as fungus growth and mycotoxin synthesis and transformation can occur in a distinct way under such conditions. Our research emphasizes how complex processes related to the activity of pathogens and their ability for biosynthesis of mycotoxins and interactions with living plant material can occur during grain malting. The analysis of their origins is key to ensuring food safety in the context of malting production, but there is still need for further research in this area.

## 4. Materials and Methods

### 4.1. Wheat Samples

The grains of 6 cultivars of winter wheat (KWS Ozon, Legenda, Muszelka, Pokusa, Sailor, and Tonacja) were selected considering their different susceptibility of wheat genotypes to Fusarium Head Blight (FHB) [37]. Each cultivar of winter wheat grown in 2018 on test plots was inoculated with *F. culmorum* (1.0 × 10^6^ spores per 1 mL) during a flowering stage (65–69 on the BBCH-scale). Winter wheat samples were collected during harvest and stored at a low temperature (4 °C) until the malting process. The grains and fungi were supplied from the resources of Poznan University of Life Sciences.

### 4.2. Toxigenic Potential of Fusarium Strain

*F. culmorum* KF-846 strain previously isolated from infected wheat was grown on 90 mm plates (Schott, Jena, Germany) with potato dextrose agar (PDA) medium (Oxoid, Basingstoke, Hampshire, UK) for 7 days at room temperature in the dark. The identity of the strain was confirmed using molecular identification by rtPCR, according to Góral et al. [38].

For toxin quantification, sterile rice cultures were prepared in triplicate according to Gálvez et al. [39]. White rice grain (50 g per 300 mL flask (ChemLand, Stargard, Poland) with 12.5 mL of distilled water) was left overnight and sterilized by means of autoclaving the next day (121 °C, 20 min). The flasks were inoculated with 4 cm^2^ of 7-day-old mycelia of the strain grown on PDA medium. The culture humidity was maintained at approximately 30% for 14 days. The cultures were then dried at room temperature and analyzed.

### 4.3. Standards and Reagents

Certified analytical standards for DON, NIV, DON-3G, ZEN, α-ZEL, and β-ZEL were purchased from Romer Labs (Tulln, Austria). The certified standards of ZEN-14G and ZEN-14S were purchased from Aokin (Berlin, Germany). NIV-3G was isolated from wheat and identified using NMR spectroscopy according to the procedure described by Yoshinari et al. [40]. ERG and 7-dehydroxycholesterol (7-DHC) standards were purchased from Sigma-Aldrich (St. Louis, MO, USA). Other reagents (formic acid, ammonium formate, leucine, enkephalin) and liquid chromatography solvents (acetonitrile, methanol, 2-propanol) were purchased from Witko (Łódź, Poland).

### 4.4. Malting

Malting of wheat samples was conducted in triplicate in the IBPRS Beer and Malt Laboratory in accordance with 1.5.3 MEBAK (Mitteleuropäische Brautechnische Analysenkommission). Portions of wheat grain (approximately 600 g each) were placed in baskets with a perforated bottom and subjected to alternating cycles of dipping and aeration using the following schedule: 7 h of steeping, 17 h of aeration, followed by 7 h of steeping with a new portion of water. During the steeping and germination of grain, the temperature was maintained at 14 °C, with the relative air humidity at a minimum of 95%. After completion of the process, the humidity of the samples was maintained at 44% by spraying with water. From that moment, the grain germination process was conducted for 7 days, with stirring once a day. After 7 days, the malt was dried for 24 h; for the first 18 h, the temperature was maintained at 45 °C, following which it was increased to 80 °C.

### 4.5. Sample Preparation—Mycotoxin Analysis

During the malting process, from the start of germination, every 48 h, approximately 50 g of each sample was taken and frozen at −30 °C. The samples were lyophilized in an Alpha 1–4 LSCplus freeze dryer (Martin Christ, Osterode am Harz, Germany) for 16 h using a shelf temperature of 25 °C and then milled in a Grindomix GM 200 knife mill (Retsch, Haan, Germany) at 10,000 rpm for 15 s. The analytical procedure was conducted as described by Nathanail et al. [41]. The sample (0.5 g) was combined with 0.1 g of NaCl and homogenized with 2 mL of extraction liquid (acetonitrile: water: formic acid, 79:20:1, *v*/*v*/*v*) in a MM 400 ball mill (Retsch, Haan, Germany) for 4 min at 30 rpm. The extract was filtered through a 0.45 µm syringe filter (Macherey-Nagel, Duren, Germany) and 350 µL of the filtrate was evaporated under a stream of nitrogen. The residue was dissolved in 700 µL of 50% methanol. The sample was filtered through a 0.22 µm syringe filter (Macherey-Nagel) and analyzed as three independent replicates.

### 4.6. LC-MS Analysis of Mycotoxins

The samples were analyzed using an H-class liquid chromatograph coupled to a mass spectrometer with a time-of-flight analyzer (UPLC-TOF-HRMS; Waters, Milford, MA, USA). Five microliters of samples were separated using a UPLC C18 Cortecs (2.1 × 100 mm, 1.6 μm) column with an appropriate pre-column (both Waters). The mobile phases were 90:10 (*v*/*v*) methanol-water (phase A) and 10:90 (*v*/*v*) methanol-water (phase B) both containing 0.2% formic acid and 20 mM ammonium formate. The flow gradient was: 0–2 min, 100% B; 3–6 min, 50% B; 22–23 min, 100% A; 25–28 min, 100% B; the flow rate was 0.3 mL/min. The mass spectrometer was operated in the negative electrospray ionization mode, with an ion source temperature of 150 °C and desolvation temperature of 350 °C. The nebulizing gas (N_2_) flow rate was 750 L/min, while the cone gas flow rate was 40 L/min. The capillary bias was 3.2 kV. The mass range of the analysis was 200–600 *m*/*z*, and the scan speed was 0.1 s. Ion optics was operated in V mode and the instrument was calibrated using leucine-enkephalin solution. The presented method allowed simultaneous analysis of three major mycotoxins abundant in wheat and six of their masked forms; the identity of analytes was confirmed by the mass accuracy.

### 4.7. Ergosterol Quantification

ERG content was determined using a modified method proposed by Perkowski et al. [42]. A lyophilized and ground wheat or malt sample (0.25 g) was shaken for 2 min in a falcon tube with 2 mL of methanol, 2 mL of 2-propanol, and addition of an internal standard (7-DHC). Following that, 1 mL of 2 M aqueous potassium hydroxide was added to the sample, and it was shaken again. The samples were heated for 2 min in a beaker with boiling water and cooled for 5 min on ice. The boiling and cooling processes were repeated 3 times. The cooled samples were neutralized with 1 mL of 1 M aqueous hydrochloric acid and 2 mL of deionized water and extracted with hexane (3 × 3 mL). The combined hexane extracts were evaporated to dryness under a nitrogen stream. Prior to analysis, the samples were dissolved in 5 mL of methanol, followed by filtration through a 0.45 µm syringe filter, and HPLC analysis. The chromatographic analyses were conducted using a Sunfire C18, 5 μm, 4.6 mm × 250 mm analytical column (Macherey-Nagel). Separation of the 45 μL samples was performed within 20 min at a flow rate of 1 mL/min and a column temperature of 40 °C. The samples were eluted using an isocratic flow of methanol-acetonitrile (9:1 *v*/*v*). The compounds were quantified by measuring the UV absorption at 282 nm; their retention times were 10.5 min (ERG) and 11.1 min (7-DHC).

### 4.8. Statistical Analysis and Software

All analyzes of individual samples were performed in triplicates. Statistical analysis was performed using one-way analysis of variance (ANOVA), while the significance of the differences was determined using the Games Howell test (α = 0.05).

The LC-MS system was managed using Waters MassLynx (Waters, Miford, MA, USA), while HPLC using EuroChrom2000 (Knauer, Berlin, Germany). For the statistical analysis, Statistica 7 (Statsoft, Tulsa, OK, USA) and MS Excel 365 (Microsoft, Redmond, WA, USA) were used.

### 4.9. Validation of Methods

The description of the validation of the methods is presented in the Supplementary Materials. Its results are shown in Tables S1–S3.

## Figures and Tables

**Table 1:** Content of mycotoxins and ergosterol in the following stages of malting of grain of six wheat cultivars. The concentrations of α-ZEL and β-ZEL are in µg/kg, while the concentrations of other mycotoxins and ergosterol in mg/kg. The different letters denote statistical significance between days of malting; the malting process was performed in triplicate.

Cultivar	KWS Ozon					Legenda				
Day of Malting	0	1	3	5	7	0	1	3	5	7
DON	7.98 ± 0.84 a	4.11 ± 0.38 b	6.45 ± 0.53 bc	7.67 ± 1.42ac	11.84 ± 1.33 d	4.72± 0.80 a	1.46± 0.17 b	2.99 ± 0.41 c	5.12 ± 0.82 a	10.94 ± 0.76 d
DON-3G	1.10 ± 0.11 a	0.85 ± 0.25 a	1.29 ± 0.46 a	3.05 ± 0.67 b	5.92 ± 0.12 c	0.43± 0.06 a	0.39± 0.05 a	1.39 ± 0.25 b	2.66 ± 0.49 c	4.30 ± 0.45 d
NIV	≤LOQ	≤LOQ	≤LOQ	≤LOQ	≤LOQ	0.89± 0.03 a	0.52± 0.076 b	0.63 ± 0.08 b	1.06 ± 0.05 c	1.20 ± 0.10 c
NIV-3G	≤LOQ	≤LOQ	≤LOQ	≤LOQ	≤LOQ	0.31± 0.01 a	0.42± 0.04 b	0.56 ± 0.07 c	0.69 ± 0.04 d	0.87 ± 0.07 e
ZEN	0.69 ± 0.03 a	0.21 ± 0.01 b	0.83 ± 0.03 c	1.52 ± 0.12 d	4.60 ± 0.06 e	0.13± 0.01 a	0.02± 0.00 b	0.07 ± 0.00 c	0.49 ± 0.03 d	0.82 ± 0.03 e
ZEN-14S	3.39 ± 0.42 a	1.85 ± 0.05 b	5.05 ± 0.49 c	7.87 ± 1.59 d	11.84 ± 0.15 e	0.83± 0.13 a	0.07± 0.01 b	0.61 ± 0.03 c	1.08 ± 0.02 d	1.66 ± 0.12 e
ZEN-14G	≤LOQ	≤LOQ	≤LOQ	≤LOQ	≤LOQ	≤LOQ	≤LOQ	≤LOQ	≤LOQ	≤LOQ
α-ZEL	≤LOQ	≤LOQ	≤LOQ	≤LOQ	≤LOQ	≤LOQ	≤LOQ	≤LOQ	≤LOQ	≤LOQ
β-ZEL	54.1 ± 5.1 a	21.2 ± 2.2 b	50.7 ± 16.9 a	58.3 ± 4.0 a	113.7 ± 7.9 c	10.3± 2.1 a	≤LOQ	≤LOQ	≤LOQ	19.9 ± 2.1 b
ERG	30.4 ± 2.6 ab	16.6 ± 0.9 c	26.0 ± 2.1 a	35.9 ± 1.4 b	57.5 ± 2.2 d	20.0± 1.3 a	8.9± 0.4 b	10.4 ± 0.6 b	15.9 ± 4.3 a	22.1 ± 0.9 a
cultivar	Muszelka					Pokusa				
day of malting	0	1	3	5	7	0	1	3	5	7
DON	9.59 ± 0.50 ab	3.42 ± 0.17 c	6.63 ± 1.18 d	8.02 ± 0.78 ad	11.77 ± 0.34 b	2.49± 0.23 a	1.83± 0.30 a	3.53 ± 0.35 b	4.47 ± 0.63 c	6.53 ± 0.92 d
DON-3G	1.24 ± 0.13 a	0.48 ± 0.01 b	1.20 ± 0.20 a	2.15 ± 0.39 c	4.07 ± 0.03 d	0.50± 0.01 a	0.88± 0.10 b	1.36 ± 0.20 c	1.84 ± 0.34 d	3.50 ± 0.22 e
NIV	≤LOQ	≤LOQ	≤LOQ	≤LOQ	≤LOQ	≤LOQ	≤LOQ	≤LOQ	≤LOQ	≤LOQ
NIV-3G	≤LOQ	≤LOQ	≤LOQ	≤LOQ	≤LOQ	≤LOQ	≤LOQ	≤LOQ	≤LOQ	≤LOQ
ZEN	0.79 ± 0.01 a	0.17 ± 0.01 b	2.58 ± 0.04 c	5.76 ± 0.18 d	9.11 ± 1.09 e	0.28± 0.02 a	0.14± 0.02 b	0.23 ± 0.01 c	1.74 ± 0.10 d	2.39 ± 0.19 e
ZEN-14S	3.64 ± 0.17 a	1.47 ± 0.11 b	4.75 ± 1.09 a	9.80 ± 0.54 c	14.35 ± 0.88 d	1.78± 0.17 a	0.43± 0.03 b	0.60 ± 0.02 c	1.81 ± 0.05 a	3.73 ± 0.57 d
ZEN-14G	≤LOQ	≤LOQ	≤LOQ	≤LOQ	≤LOQ	≤LOQ	≤LOQ	≤LOQ	≤LOQ	≤LOQ
α-ZEL	≤LOQ	≤LOQ	≤LOQ	≤LOQ	≤LOQ	≤LOQ	≤LOQ	≤LOQ	≤LOQ	≤LOQ
β-ZEL	62.2 ± 7.1 a	21.1 ± 2.1 b	89.0 ± 4.7 a	142.1 ± 13.4 c	181.9 ± 28.6 c	23.4± 1.4 a	9.1± 2.0 b	14.5 ± 0.7 c	46.2 ± 2.4 d	52.8 ± 4.0 d
ERG	44.8 ± 1.3 a	35.5 ± 1.0 b	40.0 ± 1.1 b	46.9 ± 4.9 a	69.9 ± 3.7 c	14.6± 0.5 a	8.2± 0.4 b	11.3 ± 0.1a	17.9 ± 0.6 c	22.5 ± 4.4 d
cultivar	Sailor					Tonacja				
day of malting	0	1	3	5	7	0	1	3	5	7
DON	5.08 ± 0.18 a	2.53 ± 0.39 b	3.79 ± 0.27 c	4.53 ± 0.25 d	8.37 ± 0.16 e	4.64 ± 0.38 a	1.29 ± 0.18 b	2.99 ± 0.45 c	5.72 ± 1.17 a	8.09 ± 0.65 d
DON-3G	0.86 ± 0.17 a	0.56 ± 0.06 a	1.18 ± 0.13 b	1.54 ± 0.14 c	3.04 ± 0.31 d	0.57 ± 0.05 a	0.35 ± 0.06 b	1.04 ± 0.21 c	1.28 ± 0.20 c	3.25 ± 0.60 d
NIV	≤LOQ	≤LOQ	≤LOQ	≤LOQ	≤LOQ	≤LOQ	≤LOQ	≤LOQ	≤LOQ	≤LOQ
NIV-3G	≤LOQ	≤LOQ	≤LOQ	≤LOQ	≤LOQ	≤LOQ	≤LOQ	≤LOQ	≤LOQ	≤LOQ
ZEN	0.20 ± 0.04 a	0.07 ± 0.01 b	0.25 ± 0.01 a	0.51 ± 0.05 c	1.78 ± 0.14 d	0.19 ± 0.02 a	0.03 ± 0.02 b	0.15 ± 0.01 a	0.54 ± 0.02 c	1.82 ± 0.45 d
ZEN-14S	0.60 ± 0.05 a	0.31 ± 0.01 b	0.66 ± 0.03 a	0.90 ± 0.12 c	2.61 ± 0.29 d	1.11 ± 0.16 a	0.21 ± 0.03 b	0.57 ± 0.02 c	1.65 ± 0.12 d	2.91 ± 0.32 e
ZEN-14G	≤LOQ	≤LOQ	≤LOQ	≤LOQ	≤LOQ	≤LOQ	≤LOQ	≤LOQ	≤LOQ	≤LOQ
α-ZEL	≤LOQ	≤LOQ	≤LOQ	≤LOQ	≤LOQ	≤LOQ	≤LOQ	≤LOQ	≤LOQ	≤LOQ
β-ZEL	18.6 ± 2.3 a	≤LOQ	21.3 ± 3.8 a	29.7 ± 2.4 b	40.9 ± 5.4 c	20.2 ± 5.0 a	≤LOQ	≤LOQ	14.4 ± 0.8 a	32.7 ± 1.7 b
ERG	16.6 ± 0.6 a	8.5 ± 1.1 b	12.9 ± 0.6 b	19.8 ± 1.1 c	20.9 ± 1.4 c	26.2 ± 2.2 ac	16.9 ± 1.3 b	20.2 ± 1.6 bc	23.6 ± 0.2 ac	24.5 ± 0.4 ac

The days of malting: “0”—raw grain, “1”—grain after steeping, “3”—grain after 48 h of germination, “5”—grain after 96 h of germination, “7”—grain after 144 h of germination (finished malt). Abbr.: DON—deoxynivalenol, DON-3G—deoxynivalenol-3-glucoside, NIV—nivalenol, NIV-3G—nivalenol-3-glucoside, ZEN—zearalenone, ZEN-14S—zearalenone-14-sulfite, ZEN-14G—zearalenone-14-glucoside, α-ZEL—α-zearalenol, β-ZEL—β-zearalenol, ERG—ergosterol.

**Table 2:** Relative changes in mycotoxins and ergosterol content (in%) during malting of the six wheat cultivars. Asterisks denote statistically significant changes.

Cultivar	KWS Ozon					Legenda				
Day of Malting	1 vs. 0	3 vs. 1	5 vs. 1	7 vs. 1	7 vs. 0	1 vs. 0	3 vs. 1	5 vs. 1	7 vs. 1	7 vs. 0
DON	−49 *	+57	+87 *	+188 *	+48 *	−69 *	+104 *	+251 *	+649 *	+132 *
DON−3G	−23	+52	+259 *	+596 *	+437 *	−8	+255 *	+581 *	+999 *	+912 *
NIV	−	−	−	−	−	−42 *	+22	+104 *	+130 *	+34 *
NIV−3G	−	−	−	−	−	+36 *	+33 *	+64 *	+107 *	+183 *
ZEN	−70 *	+301 *	+631 *	+2110 *	+564 *	−81 *	+175 *	+1921 *	+3329 *	+538 *
ZEN−14S	−45 *	+171 *	+325 *	+539 *	+249 *	−92 *	+790 *	+1488 *	+2343 *	+100 *
β−ZEL	−61 *	+139 *	+175 *	+436 *	+110 *	−	−	−	−	+93 *
ERG	−45 *	+57 *	+116 *	+246 *	+89 *	−56 *	+17	+79 *	+148 *	+11
cultivar	Muszelka					Pokusa				
day of malting	1 vs. 0	3 vs. 1	5 vs. 1	7 vs. 1	7 vs. 0	1 vs. 0	3 vs. 1	5 vs. 1	7 vs. 1	7 vs. 0
DON	−64 *	+94 *	+135 *	+244 *	+23	−27	+93 *	+145 *	+258 *	+163 *
DON−3G	−61% *	+150 *	+349 *	+747 *	+229 *	+77 *	+55 *	+108 *	+296 *	+601 *
ZEN	−78 *	+1411 *	+3267 *	+5226 *	+1059 *	−51 *	+67 *	+1172 *	+1641 *	+749 *
ZEN−14S	−60 *	+223 *	+566 *	+875 *	+294 *	−86 *	+40 *	+326 *	+777 *	+110 *
β−ZEL	−66 *	+322 *	+573 *	+762 *	+192 *	−61 *	+59 *	+408 *	+480 *	+126 *
ERG	−21 *	+13	+32 *	+97 *	+56 *	−44 *	+38 *	+118 *	+174 *	+54 *
cultivar	Sailor					Tonacja				
day of malting	1 vs. 0	3 vs. 1	5 vs. 1	7 vs. 1	7 vs. 0	1 vs. 0	3 vs. 1	5 vs. 1	7 vs. 1	7 vs. 0
DON	−50 *	+49 *	+79 *	+230 *	+65 *	−72 *	+131 *	+342 *	+526 *	+75 *
DON−3G	−35	+109 *	+173 *	+441 *	+252 *	−40 *	+200 *	+270 *	+842 *	+466 *
ZEN	−65 *	+262 *	+632 *	+2504 *	+808 *	−84 *	+407 *	+1683 *	+5960 *	+877 *
ZEN−14S	−48 *	+113 *	+192 *	+751 *	+339 *	−81 *	+171 *	+686 *	+1285 *	+163 *
β−ZEL	−	−	−	−	+120 *	−	−	−	−	+62 *
ERG	−49 *	+52 *	+133 *	+146 *	+26 *	−35 *	+20	+40 *	+45 *	−6

The days of malting: “0”—raw grain, “1”—grain after steeping, “3”—grain after 48 h of germination, “5”—grain after 96 h of germination, “7”—grain after 144 h of germination (finished malt). Abbr.: DON—deoxynivalenol, DON-3G—deoxynivalenol-3-glucoside, NIV—nivalenol, NIV-3G—nivalenol-3-glucoside, ZEN—zearalenone, ZEN-14S—zearalenone-14-sulfite, β-ZEL—β-zearalenol, ERG—ergosterol.

## Supplementary Materials

The following are available online at https://www.mdpi.com/article/10.3390/toxins13120866/s1, Table S1. Validation of mycotoxin analysis method: linearity, sensitivity, and separation parameters, Table S2. Validation of mycotoxin analysis method: recovery and repeatability, Table S3. Validation of ergosterol analysis method: recovery and repeatability.
