# Supplementary material for: Transformation of Selected Fusarium Toxins and Their Masked Forms during Malting of Various Cultivars of Wheat
Source: Toxins (Basel). 2021 Dec 4;13(12):866. doi: 10.3390/toxins13120866 (PMC8707366; doi:10.3390/toxins13120866)
Supplement: Supplementary file 1 [file toxins-13-00866-s001.zip › toxins-1449001-supplementary.pdf]

Supplementary Materials: Transformation of selected *Fusarium* toxins and their masked forms during malting of various cultivars of wheat

Edyta Ksieniewicz-Woźniak, Marcin Bryła, Dorota Michałowska, Agnieszka Waśkiewicz and Tomoya Yoshinari

1. Validation of methods

1.1. Mycotoxins

To validate the method, mycotoxin solutions were used to prepare calibration curves in the range of 10–1000 μg/kg (α-ZEL and β-ZEL) and 100–10,000 μg/kg for other mycotoxins. In the UPLC-MS technique, due to the influence of the matrix, which may affect the analytical signal (attenuation or amplification of the ion signal), the calibration solutions were prepared in the matrix using blank samples. These samples were prepared in the same manner as the test samples (Section 2.5 of the manuscript), and the standards were added at the final stage. The calibration curves for each analyte were characterized by coefficient of determination (R^2) values above 0.97 (Table S1). The limits of detection and quantification were estimated based on the analysis of standard solutions, where the signal-to-noise values were 3 and 10, respectively. As part of the validation experiment, the recovery of individual analytes and repeatability were expressed in as relative standard deviation (RSD). The values obtained during analysis of three independent samples are presented in Table S2. The tests used blank samples (wheat and malt) to which the test analytes were added at appropriate gain levels corresponding to the range of the standard curve and the expected levels in the test samples, following which they were analyzed in the same way as the test samples (Sections 4.5 and 4.6 of the manuscript). The validation experiment resulted in the recovery values ranging from 77.1% to 103.2%, depending on the test analyte and fortification level, with the repeatability (expressed as RSD) in most cases not exceeding 10%. The accuracy of the method was examined in the context of the method effectiveness criteria listed in Regulation (EC) No. 401/2006. In case of DON and ZEN, the recovery should be in the range of 70-120%, while the RSD should not exceed 20% and 25%, respectively. This regulation does not define criteria for NIV and modified forms of mycotoxins; however, when effectiveness criteria from DON and ZEN were adopted, the method used met the above-mentioned requirements (Table S2).

Table S1. Validation of mycotoxin analysis method: linearity, sensitivity, and separation parameters.

Analyte	Ion mass (m/z)	Retention time (min)	LOD (µg/kg)	LOQ (µg/kg)	Coefficient of determination (R^2)
DON	341.183 $(M + FA\text{-}H)^-$	4.08	20.0	60.0	0.9976
DON-3G	503.186 $(M + FA\text{-}H)^-$	4.22	12.0	36.0	0.9974
NIV	357.187 $(M + FA\text{-}H)^-$	2.38	20.0	60.0	0.9324
NIV-3G	519.190 $(M + FA\text{-}H)^-$	2.45	12.0	36.0	0.9719
ZEN	317.089 $(M\text{-}H)^-$	11.65	6.0	18.0	0.9958
ZEN-14S	397.089 $(M\text{-}H)^-$	7.25	10.0	30.0	0.9941
ZEN-14G	525.114 $(M + FA\text{-}H)^-$	6.63	10.0	30.0	0.9981
α-ZEL	319.089 $(M\text{-}H)^-$	10.75	2.5	7.5	0.9838
β-ZEL	319.089 $(M\text{-}H)^-$	8.95	2.5	7.5	0.9887

Abbr.: LOD—limit of detection, LOQ—limit of quantification, DON—deoxynivalenol, DON-3G—deoxynivalenol-3-glucoside, NIV—nivalenol, NIV-3G—nivalenol-3-glucoside, ZEN—zearalenone, ZEN-14S—zearalenone-14-sulfite, ZEN-14G—zearalenone-14-glucoside, α-ZEL—α-zearalenol, β-ZEL—β-zearalenol, M -parent molecule, FA – formic acid, H -hydrogen.

Table S2. Validation of mycotoxin analysis method: recovery and repeatability.

Analyte	Fortification (µg/kg)	Grain		Malt	
		RSD (%)	R (%)	RSD (%)	R (%)
DON	100	6.9	95.3	4.7	102.7
	500	4.8	86.1	5.2	94.3
	2500	1.7	84.3	3.0	96.7
	10000	1.2	85.1	2.4	91.8
DON-3G	100	7.2	103.2	7.1	101.2
	500	6.2	100.3	2.9	97.4
	2500	5.1	102.0	3.6	100.0
	10000	2.6	96.4	5.2	92.0
NIV	100	4.3	91.0	4.8	93.7
	500	3.8	93.2	7.6	96.6
	2500	6.0	91.3	5.0	88.4
	10000	3.1	87.4	3.1	90.6
NIV-3G	100	9.2	92.4	4.9	83.2
	500	6.1	88.2	3.3	88.4
	2500	7.4	88.6	6.0	86.4
	10000	2.1	85.6	6.0	87.7
ZEN	100	8.2	87.3	3.8	79.3
	500	4.3	92.1	4.1	81.4
	2500	5.1	96.3	4.5	87.2
	10000	3.3	94.3	3.1	85.1
ZEN-14S	100	7.4	88.4	4.2	83.3
	500	3.6	83.2	7.1	80.5
	2500	4.4	87.1	4.8	80.9
	10000	5.1	90.2	7.0	81.5
ZEN-14G	100	15.2	87.4	8.1	80.0
	500	9.6	88.1	4.3	83.2
	2500	6.3	82.0	7.0	85.6
	10000	7.0	85.5	3.5	81.5
α-ZEL	10	17.5	80.3	7.3	85.3
	50	11.4	77.4	11.0	82.4
	250	12.7	83.2	4.8	80.4
	1000	8.4	88.8	5.6	84.0
β-ZEL	10	14.8	82.6	12.1	77.1
	50	13.3	80.3	9.0	89.6

	250	9.1	87.5	4.7	90.1
	1000	6.2	92.4	6.3	86.4

Abbr.: RSD—relative standard deviation, R—recovery, DON—deoxynivalenol, DON-3G—deoxynivalenol-3-glucoside, NIV—nivalenol, NIV-3G—nivalenol-3-glucoside, ZEN—zearalenone, ZEN-14S—zearalenone-14-sulfite, ZEN-14G—zearalenone-14-glucoside, α-ZEL—α-zearalenol, β-ZEL—β-zearalenol.

1.2. Ergosterol

The linearity of the method was evaluated using a 5-point standard curve in the range of 2–100 mg/kg. The coefficient of determination (R^2) value was 0.9998. The recovery (R) and repeatability (RSD) of the method were estimated from the triplicate analysis of fortified wheat and malt samples at three levels (2, 10, and 50 mg/kg) – the results are presented in Table S3.

Table S3. Validation of ergosterol analysis method: recovery and repeatability.

**Analyte**	**Fortification (mg/kg)**	**Grain**		**Malt**	
		**RSD (%)**	**R (%)**	**RSD (%)**	**R (%)**
ergosterol	2	6.9	95.9	7.6	101.2
	10	3.1	96.1	5.0	97.3
	50	2.6	99.0	3.2	98.7
